# Supplementary material for: Elevated plasma cotinine is associated with an increased risk of developing IBD, especially among users of combusted tobacco
Source: PLoS One. 2020 Jul 2;15(7):e0235536. doi: 10.1371/journal.pone.0235536 (PMC7332008; doi:10.1371/journal.pone.0235536)
Supplement: S1 Table — Median (25–75 percentile) for continuous variables, proportions for categorical variables. (DOCX) [file pone.0235536.s001.docx]

| **S1 Table**. Baseline characteristics for subjects who later developed ulcerative colitis and Crohn’s disease (cases) and matched controls. Median (25-75 percentile) for continuous variables, proportions for categorical variables. | | | | |
| --- | --- | --- | --- | --- |
| **Ulcerative colitis** | | | | |
|  | **Case** | **Control** | **p-value*** | **n Case/Control** |
| Age, years | 50.1 (40.0-59.8) | 50.1 (40.1-59.7) | 0.86 | 70/139 |
| Time to diagnosis, years | 5.26 (2.66-7.23) | n.a. | n.a. | 70/n.a. |
| Gender, women (%) | 61.4 | 54.7 | 0.77 | 70/139 |
| BMI, kg/m^2^ | 25.0 (23.2-27.5) | 25.6 (23.1-27.8) | 0.82 | 70/138 |
| Smoking (%) | 30.0 | 20.1 | 0.16 | 65/128 |
| Number of cigarettes/day (%) |  |  |  | 62/123 |
| 0 | 65.7 | 71.9 |  | 46/100 |
| 1-4 | 5.7 | 2.2 |  | 4/3 |
| 5-14 | 12.9 | 10.8 |  | 9/15 |
| 15-25 | 4.3 | 3.6 | 0.52 | 3/5 |
| Snuff use (%) | 20.0 | 13.7 | 0.46 | 65/119 |
| Cotinine, nmol/L | 11.3 (1.52-1415) | 1.43 (0.0-774) | **0.001** | 69/138 |
| Cotinine categories (%) |  |  |  | 69/138 |
| Non-tobacco users | 51.4 | 70.5 |  | 36/98 |
| Tobacco users | 47.1 | 28.8 | **0.012** | 26/52 |
| Cotinine subcategories (%) |  |  |  | 69/138 |
| Non-tobacco users | 48.6 | 66.2 |  | 34/92 |
| Passive tobacco users | 2.9 | 4.3 |  | 2/6 |
| Tobacco users | 31.4 | 20.1 |  | 22/28 |
| Heavy tobacco users | 15.7 | 8.6 | 0.064 | 11/12 |
| Cotinine, nmol/L (by type of tobacco) |  |  |  |  |
| Smokers | 1085 (504.5-1480) | 1235 (693.0-1425) | 0.55 | 16/18 |
| Snuff users | 1905 (1418-2763) | 1550 (1257-2160) | 0.38 | 10/12 |
| Smoking and snuff-use | 1750 (1268-2203) | 1710 (1120-2300) | 1.00 | 4/7 |
| Non-users | 1.69 (1.05-2.28) | 1.06 (0.00-1.87) | **0.005** | 34/81 |
| **Crohn’s disease** | | | | |
|  | **Case** | **Control** | **p-value*** | **n Case/Control** |
| Age , years | 50.2 (40.1-56.8) | 50.0 (40.2-59.7) | 0.86 | 26/52 |
| Time to diagnosis, years | 4.76 (2.50-8.08) | n.a. | n.a. | 26/n.a. |
| Gender, women (%) | 46.2 | 50.0 | 0.94 | 26/52 |
| BMI, kg/m^2^ | 26.1 (23.1-30.4) | 25.3 (22.9-28.3) | 0.43 | 26/52 |
| Smoking (%) | 34.6 | 17.3 | 0.18 | 22/42 |
| Number of cigarettes/day (%) |  |  |  | 21/41 |
| 0 | 50.0 | 67.3 |  | 13/35 |
| 1-4 | 0.0 | 3.8 |  | 0/2 |
| 5-14 | 15.4 | 7.7 |  | 4/4 |
| 15-25 | 15.4 | 0.0 | **0.013** | 4/0 |
| Snuff use (%) | 7.7 | 17.3 | 0.39 | 22/43 |
| Cotinine, nmol/L | 4.82 (0.0-1218) | 1.48 (0.0-1113) | 0.30 | 26/52 |
| Cotinine categories (%) |  |  |  | 26/52 |
| Non-tobacco users | 53.8 | 67.3 |  | 14/35 |
| Tobacco users | 46.2 | 32.7 | 0.36 | 12/17 |
| Cotinine subcategories (%) |  |  |  | 26/52 |
| Non-tobacco users | 50.0 | 55.8 |  | 13/29 |
| Passive tobacco users | 3.8 | 11.5 |  | 1/6 |
| Tobacco users | 34.6 | 21.2 |  | 9/11 |
| Heavy tobacco users | 11.5 | 11.5 | 0.48 | 3/6 |
| Cotinine, nmol/L (by type of tobacco) |  |  |  |  |
| Smokers | 1045 (756.5-1253) | 975.5 (235.4-1598) | 0.76 | 8/6 |
| Snuff users | 1370 (1370-1370) | 1325 (1213-1585) | 0.57 | 1/6 |
| Smoking and snuff-use | 2570 (2570-2570) | 2220 (627.0-n.a.) | 0.50 | 1/3 |
| Non-users | 1.26 (0.00-2.12) | 0.00 (0.00-2.07) | 0.85 | 12/27 |
| *Calculated with Mann-Whitney for continuous variables and Chi^2^ for categorical variables.  Cotinine categories – Non-tobacco users: <85 nmol/L, tobacco users: ≥85 nmol/L. Cotinine subcategories – Non-tobacco users: <5 nmol/L, passive tobacco users: 5-<85 nmol/L, tobacco users: 85-1700 nmol/L, heavy tobacco users: >1700 nmol/L.  n.a.: not applicable. | | | | |
