# Supplementary material for: Elevated plasma cotinine is associated with an increased risk of developing IBD, especially among users of combusted tobacco
Source: PLoS One. 2020 Jul 2;15(7):e0235536. doi: 10.1371/journal.pone.0235536 (PMC7332008; doi:10.1371/journal.pone.0235536)
Supplement: S2 Table — (DOCX) [file pone.0235536.s002.docx]

| **S2 Table.** Conditional logistic regression, univariable odds ratios (OR) and 95 % confidence intervals (CI) and p for trend across categories, for developing ulcerative colitis and Crohn’s disease, and categories of tobacco exposure. | | | |
| --- | --- | --- | --- |
| **Ulcerative colitis** | | | |
| **Tobacco exposure** | **OR (95%CI)** | **p trend** | **n Case/Control** |
| Number of cigarettes/day |  |  | 62/117 |
| 0 | Ref |  |  |
| 1-4 | 2.35 (0.51-10.8) |  |  |
| 5-14 | 1.45 (0.58-3.63) |  |  |
| 15-25 | 1.39 (0.32-6.12) | 0.34 |  |
| Cotinine categories |  |  | 69/136 |
| Non-tobacco users | Ref |  |  |
| Tobacco users | **2.21 (1.21-4.06)** | **0.010** |  |
| Cotinine sub-categories |  |  | 69/136 |
| Non-tobacco users | Ref |  |  |
| Passive tobacco users | 1.07(0.19-6.01) |  |  |
| Tobacco users | **2.13 (1.07-4.24)** |  |  |
| Heavy tobacco users | 2.47 (0.95-6.46) | **0.011** |  |
| **Crohn’s disease** | | | |
| **Tobacco exposure** | **OR (95%CI)** | **p trend** | **n Case/Control** |
| Number of cigarettes/day |  |  | 21/39 |
| 0 | Ref |  |  |
| 1-4 | 0.0 (0.0-1.5E+163) |  |  |
| 5-14 | 3.35 (0.59-18.8) |  |  |
| 15-25 | 148631 (0.0-1.6E+129) | **0.020** |  |
| Cotinine categories |  |  | 26/52 |
| Non-tobacco users | Ref |  |  |
| Tobacco users | 1.60 (0.66-3.85) | 0.30 |  |
| Cotinine subcategories | 1.14 (0.78-1.68) |  | 26/52 |
| Non-tobacco users | Ref |  |  |
| Passive tobacco users | 0.30 (0.03-3.50) |  |  |
| Tobacco users | 1.62 (0.55-4.79) |  |  |
| Heavy tobacco users | 0.89 (0.20-3.99) | 0.51 |  |
| Cotinine categories – Non-tobacco users: <85 nmol/L, tobacco users: ≥85 nmol/L. Cotinine subcategories – Non-tobacco users: <5 nmol/L, passive tobacco users: 5-<85 nmol/L, tobacco users: 85-1700 nmol/L, heavy tobacco users: >1700 nmol/L. | | | |
