# Supplementary material for: Elevated plasma cotinine is associated with an increased risk of developing IBD, especially among users of combusted tobacco
Source: PLoS One. 2020 Jul 2;15(7):e0235536. doi: 10.1371/journal.pone.0235536 (PMC7332008; doi:10.1371/journal.pone.0235536)
Supplement: S3 Table — (DOCX) [file pone.0235536.s003.docx]

| **S3 Table.** Conditional logistic regression, univariable odds ratios (OR) and 95 % confidence intervals (CI) for developing ulcerative colitis and Crohn’s disease, subdivided by median time from data collection to diagnosis. | | |
| --- | --- | --- |
| **Ulcerative colitis** | | |
| **Tobacco exposure** | **OR (95%CI)** | **n Case/Control** |
| All cases |  |  |
| log-Cotinine | **1.33 (1.14-1.55)** | 69/136 |
| Smoking | 1.77 (0.89-3.51) | 65/128 |
| Snuff use | 1.42 (0.61-3.29) | 65/119 |
| Data collected <5 years before diagnosis* |  |  |
| log-Cotinine | **1.24 (1.05-1.45)** | 34/68 |
| Smoking | 1.59 (0.62-4.06) | 31/62 |
| Snuff use | 0.78 (0.29-2.11) | 31/58 |
| Data collected >5 years before diagnosis* |  |  |
| log-Cotinine | **1.35 (1.09-1.68)** | 35/68 |
| Smoking | 2.04 (0.88-4.69) | 34/66 |
| Snuff use | 2.03 (0.61-6.78) | 34/61 |
| **Crohn’s disease** | | |
| **Tobacco exposure** | **OR (95%CI)** | **n Case/Control** |
| All cases |  |  |
| log-Cotinine | 1.11 (0.92-1.34) | 26/52 |
| Smoking | 2.32 (0.73-7.35) | 22/42 |
| Snuff use | 0.36 (0.07-1.90) | 22/43 |
| Data collected <5 years before diagnosis* |  |  |
| log-Cotinine | 1.14 (0.89-1.45) | 14/28 |
| Smoking | 2.12 (0.47-9.48) | 13/25 |
| Snuff use | 0.46 (0.08-2.75) | 13/26 |
| Data collected >5 years before diagnosis* |  |  |
| log-Cotinine | 1.07 (0.80-1.44) | 12/24 |
| Smoking | 2.64 (0.43-16.0) | 9/17 |
| Snuff use | 0.03 (0.0-5748) | 9/17 |
| *5.09 years, the median time before diagnosis. | | |
