# Supplementary material for: Elevated plasma cotinine is associated with an increased risk of developing IBD, especially among users of combusted tobacco
Source: PLoS One. 2020 Jul 2;15(7):e0235536. doi: 10.1371/journal.pone.0235536 (PMC7332008; doi:10.1371/journal.pone.0235536)
Supplement: S4 Table — (DOCX) [file pone.0235536.s004.docx]

| **S4 Table.** Multivariable conditional logistic regression showing odds ratios (OR) and confidence interval (CI) for ulcerative colitis and Crohn’s disease, subdivided by median time from data collection to diagnosis. | | |
| --- | --- | --- |
| **Ulcerative colitis** | | |
|  | **OR (95% CI)** | **n Case/Control** |
| All cases |  | 64/116 |
| log-Cotinine | **1.42 (1.12-1.79)** |  |
| Smoking | 0.66 (0.26-1.73) |  |
| Snuff use | 0.55 (0.19-1.57) |  |
| Data collected <5 years before diagnosis* |  | 31/58 |
| log-Cotinine | **1.78 (1.12-2.81)** |  |
| Smoking | 0.12 (0.01-1.12) |  |
| Snuff use | 0.20 (0.03-1.34) |  |
| Data collected >5 years before diagnosis* |  | 33/58 |
| log-Cotinine | 1.32 (0.95-1.83) |  |
| Smoking | 1.08 (0.34-3.40) |  |
| Snuff use | 0.78 (0.17-3.49) |  |
| **Crohn’s disease** | | |
|  | **OR (95% CI)** | **n Case/Control** |
| All cases |  | 22/42 |
| log-Cotinine | 1.23 (0.79-1.90) |  |
| Smoking | 1.65 (0.29-9.43) |  |
| Snuff use | 0.12 (0.01-1.23) |  |
| Data collected <5 years before diagnosis* |  | 13/25 |
| log-Cotinine | 1.22 (0.74-2.03) |  |
| Smoking | 1.75 (0.19-16.15) |  |
| Snuff use | 0.14 (0.01-2.02) |  |
| Data collected >5 years before diagnosis* |  | 9/17 |
| log-Cotinine | 1.21 (0.53-2.75) |  |
| Smoking | 1.53 (0.08-27.7) |  |
| Snuff use | 0.0 (0.0-.) |  |
| Three variables were included in all multivariable models: log-Cotinine, smoking and snuff use. *5.09 years, the median time before diagnosis. | | |
